# Alleviation of aluminium-induced cell rigidity by overexpression of *OsPIN2* in rice roots

**DOI:** 10.1093/jxb/eru292

**Published:** 2014-07-22

**Authors:** Daoming Wu, Hong Shen, Ken Yokawa, František Baluška

**Affiliations:** ^1^College of Resources and Environment, South China Agricultural University, Guangzhou 510642, China; ^2^Department of Plant Cell Biology, IZMB, University of Bonn, Bonn D-53115, Germany

**Keywords:** Aluminium, auxin, cell rigidity, mechanical change, *Oryza sativa* L., *OsPIN2*.

## Abstract

Al-induced cell rigidity is one of the symptoms of Al toxicity, but the mechanism by which plants tolerate this toxicity is still unclear. In this study, we found that overexpression of *OsPIN2*, an auxin transporter gene, could alleviate Al-induced cell rigidity in rice root apices. A freeze–thawing experiment showed that the Al-treated roots of wild-type (WT) plants had more damage in the epidermal and outer cortex cells than that found in lines overexpressing *OsPIN2* (OXs), and the freeze-disrupt coefficient was 2-fold higher in the former than in the latter. Furthermore, Al could induce aberrations of the cell wall–plasma membrane interface, which was more prominent in the epidermal cells of the elongation zone of the WT. Overexpressed *OsPIN2* reduced Al-induced formation of reactive oxygen species and weakened Al-induced lipid peroxidation and lignification in roots. Compared with WT, a 16.6–32.6% lower Al-triggered hemicellulose 1 accumulation was observed in root apices of OXs, and 17.4–20.5% less Al accumulated in the cell wall of OXs. Furthermore, overexpression of *OsPIN2* ameliorated the Al inhibitory effect on basipetal auxin transport and increased Al-induced IAA and proton release. Taken together, our results suggest that by decreasing the binding of Al to the cell wall and Al-targeted oxidative cellular damage, OXs lines show less Al-induced damage. By modulating PIN2-based auxin transport, IAA efflux, and cell wall acidification, lines overexpressing *OsPIN2* alleviate Al-induced cell rigidity in the rice root apex.

## Summary

Overexpression of *OsPIN2* reduces Al-induced formation of reactive oxygen species, weakens lipid peroxidation and lignification, and thus enhances Al tolerance of rice seedlings.

## Introduction

One of the most obvious symptoms of Al toxicity in plants is the rapid inhibition of root growth (Ryan *et al.*, 1993; Kochian *et al.*, 2004), which can be measured less than 30min after Al treatment (Krtková *et al.*, 2012). Al-induced inhibition of root growth results in a poor nutrient acquisition and the loss of crop yields (Kochian, 1995). Many previous papers have reported the mechanism of Al toxicity, and indicated that Al targeted and affected multiple sites of the root cells including the cell wall, plasma membrane, cytoskeleton, mitochondria, and nucleus (Matsumoto, 2000; Ma *et al.*, 2014).

Exposure to Al stress causes accumulation of cell wall polysaccharides and lignin, resulting in the typical thick and rigid cell wall in squash (Van *et al.*, 1994), wheat (Tabuchi and Matsumoto, 2001), and rice (Yang *et al.*, 2008). These Al-dependent changes in the cell wall are involved in Al-induced reduction of cell expansibility, which may be the main cause of the Al inhibition of root elongation in short-term experiments (Ma *et al.*, 2004; Zhu *et al.*, 2012). Tabuchi and Matsumoto (2001) found that a 6-h exposure to 10 µmol l^–1^ Al significantly suppressed root elongation and caused a substantial decrease in the mechanical extensibility of the cell wall in the Al-sensitive cultivar Scout 66, but not in the Al-resistant cultivar Atlas 66. Creep-extension analysis further showed that the elasticity parameters and viscosity parameters in Scout 66 were increased by 6–22% after a 3-h Al treatment, and the total extensibility was also decreased by 20–30%, whereas these parameters were slightly affected by Al in Atlas 66 (Ma *et al.*, 2004).

On the other hand, accumulating evidence demonstrates that Al-targeted oxidative stress and the binding of Al also make walls more rigid (Sasaki *et al.*, 1996; Yamamoto *et al.*, 2001, 2002; Tabuchi and Matsumoto, 2001; Ma *et al.*, 2004; Jones *et al.*, 2006). The plant hormone auxin plays an important role in not only the responses to oxidative stress (Tamás *et al.*, 2012; Krishnamurthy and Rathinasabapathi, 2013*a*), but also the distribution of Al in cells (Zhu *et al.*, 2013). For example, auxin might be the connecting link that regulates the level of reactive oxygen species (ROS) and directs the role of ROS in oxidative stress (Iglesias *et al.*, 2010; Krishnamurthy and Rathinasabapathi, 2013*b*). A recent study found that compared with the wild-type ecotype Columbia, an auxin-overproducing mutant, *yucca*, had significantly reduced cell wall Al and increased symplastic Al content, suggesting that auxin may regulate Al distribution in root cells (Zhu *et al.*, 2013). Here, an interesting question is raised: does auxin contribute to the alleviation of Al-induced cellular rigidity?

Auxin has also been known to play a crucial role in root cell elongation (Teale *et al.*, 2005). The *PIN2* gene encodes the auxin efflux transporter PIN2, which plays a pivotal role in mediating the backward (towards the root base) auxin flow in the epidermis and outer cortex cells (Blilou *et al.*, 2005; Baluška *et al.*, 2010). Kollmeier *et al.* (2000) found that Al, similarly to the inhibitors of polar auxin transport, such as 1-N-naphthyphthalamic acid (NPA) and 2,3,5-triiodobenzoic acid (TIBA), caused the inhibition of basipetal auxin transport, and thus inhibited root growth. Evidence from *Arabidopsis* further showed that this inhibitory effect of Al on auxin transport was associated with Al-blocked PIN2-mediated auxin polar transport (Shen *et al.*, 2008; Sun *et al.*, 2010), indicating that PIN2 may emerge as an Al-toxicity target of root apices (Baluška *et al.*, 2010).

Overexpressing *OsPIN2* can enhance auxin transport from shoot to root and auxin polar transport in roots (Chen *et al.*, 2012). Acid-growth theory indicates that auxin can activate plasma membrane (PM) H^+^-ATPase and facilitate H^+^ efflux into the cell wall compartment, thus softening the cell wall and initiating extension growth (Hager, 2003; Grebe, 2005). Increasing auxin transport may be associated with softening of the cell wall and enhancing cell expansion. However, the evidence is still lacking.

In this study, the responses of an *OsPIN2*-overexpressing line and its wild-type line to Al stress were measured in hydroponic experiments. Differential physiological responses of two lines were characterized.

## Materials and methods

See Supplementary material, available at *JXB* online, for details concerning methods for microscopy observations, physical properties measurement, and gene expression.

### Plant materials and growth conditions

The rice ‘Nipponbare’ (*Oryza sativa* L. ssp. Japonica cv. Nipponbare, WT) and transgenic plants overexpressing *OsPIN2* (OX1 and OX2) were used in this study. Transgenic rice seeds (Chen *et al.*, 2012) were kindly provided by Xu Guohua from Nanjing Agricultural University, China. Seeds of WT, OX1, and OX2 were surface sterilized for 30min in a 10% (v/v) H_2_O_2_ solution, washed with deionized water, soaked in deionized water at 30 °C overnight, then germinated at 30 °C in darkness for 2 d. The germinated seeds were transferred to a net floating on a 0.5 mmol l^–1^ CaCl_2_ solution (pH 4.5) for 3 d.

### Root growth experiments

3-d-old seedlings were exposed to a 0.5 mmol l^–1^ CaCl_2_ solution (pH 4.5) containing 0, 50, and 80 µmol l^–1^ AlCl_3_ for 24h. The primary root elongation was measured with a ruler before and after treatment. The change of root surface area was analysed by WinRHIZO (Regent Instrument Inc., Canada).

### Mechanical changes of root apex cells

3-d-old seedlings of WT, OX1, and OX2 were exposed to a 0.5 mmol l^–1^ CaCl_2_ solution (pH4.5) containing 0 or 50 µmol l^–1^ AlCl_3_ for 6h. Root tips (0–5mm) were excised and embedded in 5% agar, then were transversely sectioned at 3mm from apexes with a vibratome (DTK-1000, DOSAKA, Japan). The thickness of section was 80 µm.

### Freeze–thawing experiment

Five pieces of 80 µm intact sections were placed on the loading glass with a drop of deionized water and glycerin, and covered with a cover slide. To avoid the movement of sections during the process of freeze–thawing, we sealed the edge of the cover slide with neutral balsam. This also ensures that a horizontal and steady pressure within the slide can be produced when the ice melts. The section was photographed using an OLYMPUS system microscope (BX43, OLYMPUS, Japan), and this photograph was saved as ‘Section Before Treating’ (SBT). Then the slides were frozen at –20 °C overnight. After freezing, these slides were removed from the refrigerator immediately and thawed at 30 °C for 20min. Then a new photograph was taken and saved as ‘Section After Treating’ (SAT). When the structure of cells is disrupted during freeze–thawing, a shrinking section can be observed. The more seriously the cell structure was damaged, the more significantly the section shrank.

### Freeze-disrupt coefficient calculation

The area of SBT and SAT was analysed using Image J (National Institutes of Health, USA). The difference between them is named SA, which means shrinkage area after freeze–thawing experiment. Area measurements were used to calculate freeze-disrupt coefficient (FDC). FDC was calculated by SA_t_÷SA_c_, where SA_t_ is the SA mean of treatment (including WT section with Al toxicity and OX section with or without Al treatment), and SA_c_ is the SA mean of WT section without Al toxicity.

### Microstructure observation

3-d-old seedlings of WT, OX1, and OX2 were exposed to a 0.5 mmol l^–1^ CaCl_2_ solution (pH4.5) containing 0 or 50 µmol l^–1^ AlCl_3_ for 6h. Root tips (3–4mm) were excised, and fixed with 4% (w/v) glutaraldehyde and 3% (w/v) paraformaldehyde. They were washed three times with 0.1mol l^–1^ phosphate buffer (PBS, pH 7.2) and were postfixed with 1% (w/v) OsO_4_ at –4 °C for 4h. The segments were washed three times again with the same buffer before being dehydrated in an ethanol series and embedded in epoxy resin. Ultrathin sections (70nm) were cut with ultramicrotome (Leica) and viewed at 100kV in transmission electron microscope (TEM, Tecnai 12, FEI).

### Measurement of IAA efflux

3-d-old seedlings of WT, OX1, and OX2 were transferred to a 20ml tube (20 seedlings per tube) filled with 20ml of 0.5 mmol l^–1^ CaCl_2_ solution (pH4.5) containing 0 or 50 µmol l^–1^ AlCl_3_ for 6h. After treatment, the incubative solution was collected and evaporated at 40 °C to dryness. The residue was re-dissolved with 2ml deionized water, and then filtered through a 0.45 µm filter. IAA content was analysed by the Salkowski colorimetric technique as described by Glickmann and Dessaux (1995). Briefly, 1ml of Salkowski reagent, which consisted of 12g l^–1^ FeCl_3_ in a 7.9mol l^–1^ H_2_SO_4_ solution, was mixed with 1ml of the sample solution. The mixture was kept in darkness for 30min, and the absorbance measurement was made at 530nm in spectrophotometer (UV-1700, SHIMADZU, Japan).

### Visualization of pH changes

An agar gel (1%, w/v) containing 0.01% (w/v) bromocresol green was used to indicate rhizosphere acidification via colour changes. 3-d-old seedlings of WT, OX1, and OX2 were transferred to a 20ml tube (10 seedlings per tube) filled with 20ml pH indicator gel in 0.5 mmol l^–1^ CaCl_2_ solution (pH4.5) containing 0 or 50 µmol l^–1^ AlCl_3_. The roots and gel were photographed before and after 6-h treatment using a digital camera (D300S, Nikon).

## Results

### Variation of root elongation and root surface area

**Fig. 1.**
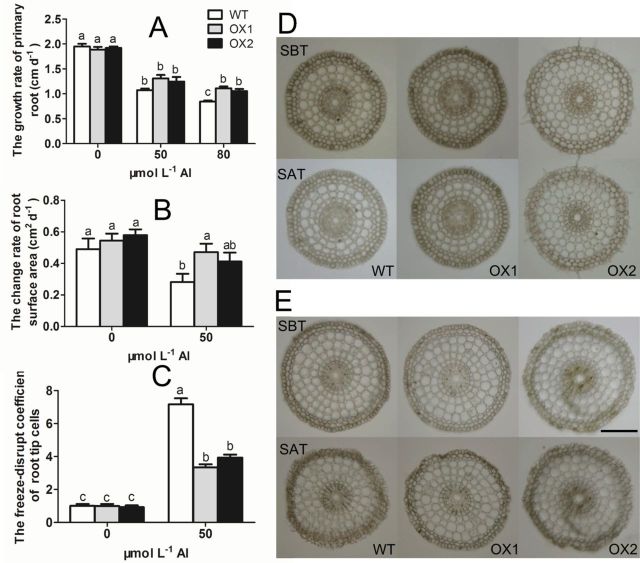
Effect of Al on root growth and the mechanical changes of root apex cells in *Nipponbare* (WT) and *OsPIN2* overexpression lines (OXs). (A) Effect of Al on primary root elongation. (B) Effect of Al on root surface area change. Values are means±SE (*n*=10). (C–E) Effect of Al on the mechanical changes of root apex cells. 80-µm root apex sections were collected and used for the freeze–thawing experiments. The change of the sections of the root tips in the presence (E) or absence (D) of 50 µmol l^–1^ AlCl_3_ were observed before (SBT) and after (SAT) the treatment, the freeze-disrupt coefficient of root apex cells was then calculated (C). Values are means±SE (*n*=10). Means with different letters are significantly different (*P*<0.05 by Tukey test). Bar=100 µm. (This figure is available in colour at *JXB* online.)

AtPIN2 was reported to be involved in the Al response (Shen *et al.*, 2008). In this study, transgenic rice lines overexpressing *OsPIN2* (OXs) and their wild type line (WT) were measured in response to Al stress. The growth rate of the primary root in different lines showed nearly no difference in Al treatments of 0 and 50 µmol l^–1^ (Fig. 1A). However, in the presence of 80 µmol l^–1^ Al, the root growth was inhibited more markedly in WT than OXs. Growth rate of the primary root of OXs was 124.6–131.7% of WT (Fig. 1A). After a 24-h treatment with 50 µmol l^–1^ AlCl_3_, the change of root surface area was also more inhibited in the WT than OXs (Fig. 1B). These results suggested that transgenic rice overexpressing *PIN2* had a higher Al tolerance than the wild-type line did.

### Mechanical changes of root apex cells

To gain insight into the Al-induced changes in mechanical properties of root apex cells, a freeze–thawing experiment was performed with root apices of rice seedlings to indicate the plasticity of cell wall. After freeze–thawing treatment, apical root sections without Al treatment remained intact (Fig. 1D), but the sections of Al-treated root were shrunk (Fig. 1E). Many epidermis and outer cortex cells were broken. Compared with OX1 and OX2, more epidermis and outer cortex cells in WT were disrupted (Fig. 1E). Subsequently, we used the freeze-disrupt coefficient (FDC) to quantify the difference. The larger the FDC was, the more serious the extent of the damage was. It was observed that the FDC of WT under Al stress was respectively 2.1 times and 1.8 times higher than that of OX1 and OX2 (Fig. 1C), suggesting that the root cells of OXs were more tolerant to Al stress than those of WT.

### Cell wall and plasma membrane microstructure

**Fig. 2.**
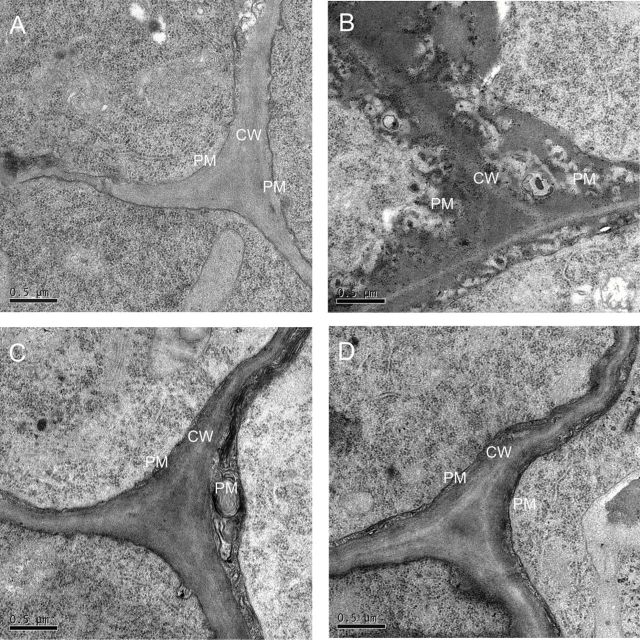
Effect of Al on the microstructure of the cell wall (CW) and plasma membrane (PM) in the epidermis cell of the root tip. Root tips (0–3mm) were excised. (A) The microstructure of CW and PM in the epidermis cell of the Al-untreated root (WT). (B–D) The microstructure of CW and PM in epidermis cell of Al-treated root (B, WT; C, OX1; D, OX2). Bar=0.5 µm.

To investigate Al-induced damage of the cell wall and plasma membrane, a microstructure experiment was performed with the Al-treated rice root apices. After a 6-h exposure to Al, the plasma membrane of the epidermis cell in the elongation zone turned clearly black, and the cell wall–plasma membrane interface became strongly convoluted (Fig. 2). These changes were more prominent in WT when compared with the cell wall–plasma membrane interface of OXs lines (Fig. 2B).

### Lipid peroxidation

**Fig. 3.**
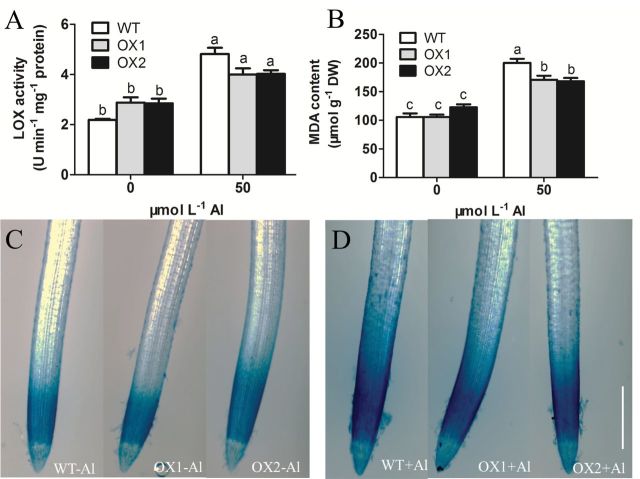
Effect of Al on the peroxidation of root tips. 3-d-old seedlings of WT, OX1, and OX2 were exposed to a 0.5 mmol l^–1^ CaCl_2_ solution (pH 4.5) containing 0 or 50 µmol l^–1^ AlCl_3_ for 6h. Root tips (0–10mm) were excised. (A) The activity of LOX. (B) The content of MDA. Values are means±SE (*n*=3). Means with different letters are significantly different (*P*<0.05 by Tukey test). (C and D) Evans blue staining; C, without Al treatment; D, with Al treatment; bar=500 µm. (This figure is available in colour at *JXB* online.)

Lipoxygenase (LOX) pathways are crucial for lipid peroxidation processes in plants; higher activity of LOX will aggravate peroxidation of the plasma membrane (Hwang and Hwang, 2010). In this study, treatment with 50 µmol l^–1^ Al enhanced LOX activity in both WT and OXs. The activity of LOX in root apices of WT was 120.1% of that of OXs (Fig. 3A).

Malondialdehyde (MDA) is the final product of lipid peroxidation, which indicates the extent of membrane peroxidation. Al treatment increased MDA content in root apices of both WT and OXs. Higher accumulation of Al-induced MDA was observed in WT than in OXs (Fig. 3B). Similarly, Al-induced Evans blue staining was enhanced in root apices (Fig. 3D). The distribution of staining pattern, especially in WT, diffused from the apical meristem zone to the elongation zone (Fig. 3D). These results indicated that more serious lipid peroxidation caused by Al stress occurred in WT than that of the OX lines.

### Reactive oxygen species (ROS)

**Fig. 4.**
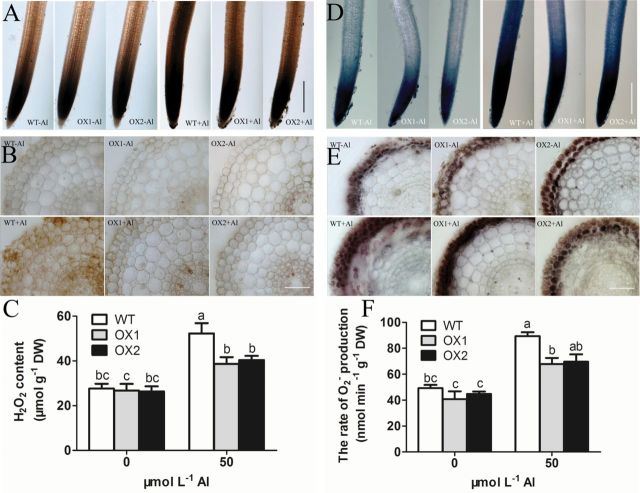
Effect of Al on ROS formation in the root apex. 3-d-old seedlings of WT, OX1, and OX2 were exposed to a 0.5 mmol l^–1^ CaCl_2_ solution (pH 4.5) containing 0 or 50 µmol l^–1^ AlCl_3_ for 6h. Root tips (0–10mm) were excised. (A) The root tips stained with DAB, bar=500 µm. (B) Section (50 µm) at 3mm from apexes stained with DAB, bar=50 µm. (C) The content of H_2_O_2_. Values are means±SE (*n*=3). (D) The root tips stained with NBT, bar=500 µm. (E) Section (50 µm) at 3mm from apexes stained with NBT, bar=50 µm. (F) The content of O_2_^–^, values are means±SE (*n*=3). Means with different letters are significantly different (*P*<0.05 by Tukey test). (This figure is available in colour at *JXB* online.)

As Al triggered changes in the cell wall and plasma membrane, and lipid peroxidation could be induced by hypergeneration of ROS (Yamamoto *et al.*, 2001, 2002; Jones *et al.*, 2006), ROS accumulation in root apexes was further investigated through histochemical staining. Nitro blue tetrazolium (NBT) could be used to detect the localization of O_2_^–^ (Dunand *et al.*, 2007), and 3,3’-Diaminobenzidin (DAB) could be used to visualize that of H_2_O_2_ (Thordal Christensen *et al.*, 1997). In this study, DAB staining was observed only in the apical meristem and transition zone (TZ) of rice roots under normal conditions. Through exposure to Al, the DAB staining was enhanced and spread to the elongation zone (EZ) in the root apices of WT, but this phenomenon was not obvious in OX1 or OX2 (Fig. 4A). A further observation of root sections revealed that most H_2_O_2_ localized in the epidermis and column cells. However, this localization became stronger and was spread to all cells after Al treatment, and this change was more significant in WT than in OXs (Fig. 4B). Quantification of H_2_O_2_ also suggested that Al toxicity increased H_2_O_2_ content in root apexes, and the Al-treated root apexes of OX1 and OX2 plants had 22.8–25.5% lower H_2_O_2_ content than that in WT (Fig. 4C).

NBT, which is a dark blue formazan dye, appeared mainly in the root cap to TZ of Al-untreated roots, but more markedly in the root cap to EZ of Al-treated roots, particularly in those of WT (Fig. 4D). Most NBT staining deposited in the epidermis and outer cortex, and these deposits were more apparent in the sections of Al-treated roots than those of Al-untreated roots (Fig. 4E). Consistent with NBT staining results, the measurement of O_2_^–^ production rate showed that Al toxicity increased O_2_^–^ formation by 81.3%, 66.6%, and 55.4% in WT, OX1, and OX2 root apices, respectively, compared with those of Al-untreated roots (Fig. 4F). These results indicated that Al increased the formation of ROS to a lesser extent in the root tips of OXs than that of WT.

### Lignin and cell wall fractions

**Fig. 5.**
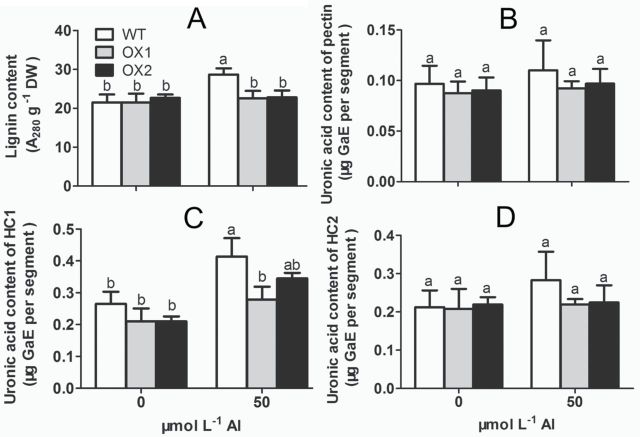
Effect of Al on lignin content and uronic acid content of cell wall fraction. 3-d-old seedlings of WT, OX1, and OX2 were exposed to a 0.5 mmol l^–1^ CaCl_2_ solution (pH 4.5) containing 0 or 50 µmol l^–1^ AlCl_3_ for 6h. Root tips (0–10mm) were excised. (A) The content of lignin. (B) Uronic acid content of pectin. (C) Uronic content of HC1. (D) Uronic acid content of HC2. Values are means±SE (*n*=3). Means with different letters are significantly different (*P*<0.05 by Tukey test).

Besides ROS, the lignin and the cell wall polysaccharides, including pectin, hemicellulose 1 (HC1), and hemicellulose 2 (HC2) were also measured under Al stress. After exposure to Al treatment, the content of lignin in WT was increased by 33.5%, whereas it was hardly affected by Al in OX1 and OX2 (Fig. 5A). The content of pectin and HC2 in the root apexes of WT and OX did not change significantly after Al treatment (Fig. 5B and 5D). However, after exposure to Al for 6h, the content of HC1 was increased by 56.2%, 32.9%, and 64.3% in WT, OX1, and OX2 root apices, respectively, and OXs had 16.6–32.6% lower HC1 content than that of WT (Fig. 5C).

### Al distribution in rice roots

**Fig. 6.**
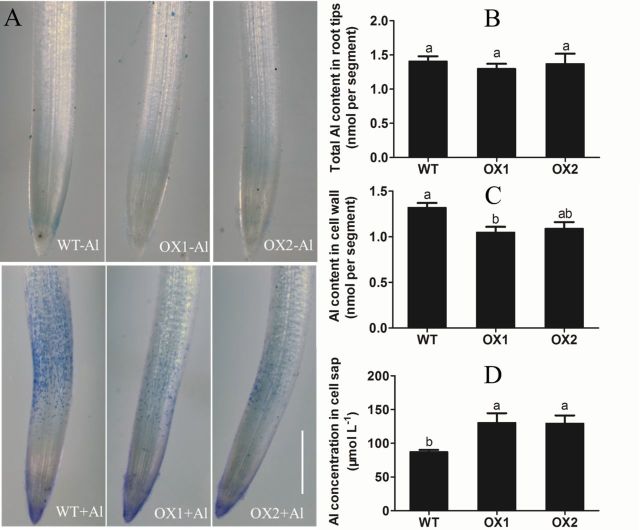
Al accumulation in WT and OXs root apexes. 3-d-old seedlings of WT, OX1, and OX2 were exposed to a 0.5 mmol l^–1^ CaCl_2_ solution (pH4.5) containing 0 or 50 µmol l^–1^ AlCl_3_ for 6h. Root tips (0–10mm) were excised. (A) The root apex stained with Eriochrome cyanine R, bar=500 µm. (B) Total Al content in root tips. (C) Al in the cell wall of root tips. (D) Al in the cell sap of root tips. Values are means±SE (*n*=3). Means with different letters are significantly different (*P*<0.05 by Tukey test). (This figure is available in colour at *JXB* online.)

Previous evidence showed that when plant roots were exposed to Al solution, most Al was bound to the cell wall of root apices, and caused the deformation of the cell wall, which would result in the break load of the cell wall (Tabuchi and Matsumoto, 2001; Ma *et al.*, 2004). In this study, Al allocation in the root cell wall and cell sap in both WT and OXs was investigated. Al content in 0–10mm root segments was quantified after an Al exposure of 6h. Results from Fig. 6 show that OXs accumulated similar levels of Al to WT in root tips; no significant differences of total Al content were observed in WT and OXs (Fig. 6B). However, significant differences of Al content in the root cell wall and cell sap were observed between WT and OX1 (Fig. 6C and 6D). The Al content in the cell wall of OX1 and OX2 was 17.4–20.5% lower than that of WT (Fig. 6C), whereas the Al concentration in cell sap was 48.6–49.8% higher in OX1 and OX2 than in the WT (Fig. 6D), indicating that a lower proportion of cell wall bound Al over root sap Al occurred in overexpression lines.

On other hand, Eriochrome Cyanine R staining, which can detect Al accumulation in the surface of the root, showed that a stronger pink colour occurred in TZ and EZ of WT than that of OX1 and OX2 (Fig. 6A), suggesting that more Al was accumulated on root surface of WT than that of OX1 and OX2 when they were exposed to Al treatment.

### Auxin concentration, rhizosphere pH, and gene expression

**Fig. 7.**
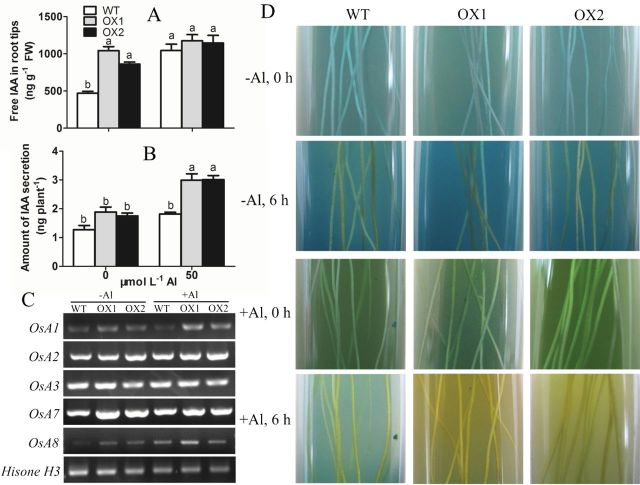
Effect of Al on the change of IAA concentration and rhizosphere pH. (A) The concentration of free IAA in root tips. (B) Amount of IAA secretion per rice seedling. Values are means±SE (*n*=3). Means with different letters are significantly different (*P*<0.05 by Tukey test). (C) Semi-quantitative RT-PCR analysis of *OsA* gene expression. *Histone H3* was used as loading control. (D) Colour changes in bromocresol green agar medium. The colour of the medium around the roots changed to yellow showing that the pH in the rhizosphere had decreased. (This figure is available in colour at *JXB* online.)

Acid-growth theory states that IAA plays an important role in cell wall loosening by activating PM H^+^-ATPase and raising the proton concentration in the cell wall compartment (Hager, 2003). To investigate the cause of cell wall loosening, auxin concentration and rhizosphere pH were measured in root tips of both WT and OXs. Overexpression of *OsPIN2* had a 1.5–2.5-fold increase of free IAA concentration in root apices in comparison to WT under normal condition (Fig. 7A). Al caused an increase in the free IAA concentration in the root apexes of WT and OXs. Interesting, this effect of Al was much more prominent in WT compared with OXs. Particularly, Al increased the free IAA concentration by 115.4% in WT, but only by 12.9% and 33.0% in OX1 and OX2, respectively (Fig. 7A).

PM H^+^-ATPase, which is encoded by *OsAHA* or *OsA*, mediates ATP-dependent proton extrusion to the extracellular space (Baxter *et al.*, 2003; Chang *et al.*, 2009). Expression analyses of *OsA* showed that overexpression of *OsPIN2* up-regulated *OSA1*, *OSA2*, *OSA3*, *OSA7*, and *OSA8* (Fig. 7C). In the presence of Al, only the expression level of *OsA8* was up-regulated in WT, whereas the expression levels of both *OsA1* and *OsA8* were up-regulated in OXs (Fig. 7C), indicating that Al treatment might result in an higher increase in PM H^+^-ATPase activity in OXs than in WT.

We subsequently analysed the changes in rhizosphere pH via *in situ* visualization. The yellow colour due to proton extrusion was significantly visualized in the indicator gel with OX1 or OX2 seedlings under the condition of 50 µmol l^–1^ Al (Fig. 7D). This finding suggested that overexpressing *OsPIN2* could increase Al-induced proton exudation from rice roots.

To determine whether the difference of Al-induced proton extrusion that occurred in the roots was associated with the exudation of IAA, we tried to collect the secretion of IAA and quantify it by using the Salkowski colorimetric method (Glickmann and Dessaux, 1995). A small amount of IAA was detected in the root exudates in the absence of Al. However, after exposure to 50 µmol l^–1^ Al, the IAA exudation of WT, OX1, and OX2 was increased by 42.9%, 135.4%, and 136.2%, respectively, in comparison to WT without Al treatment (Fig. 7B), and this change showed a similar pattern to the change of rhizosphere pH (Fig. 7D).

## Discussion

After a short-time freeze, the sap both in the apoplast and symplast is converted into ice crystals. These ice crystals can be reversibly thawed when the temperature rises. During a quick freeze–thawing process, the PM and the cell wall (CW) will be rapidly impaired by ice crystal damage and turgor pressure, thus disrupting the structure of the cell (Burke *et al.*, 1976; Pearce and Fuller, 2001; Gusta and Wisniewski, 2013). When the freeze–thawing conditions stay stable, this disruption is mainly dependent on the mechanical properties of the cell, such as the elastic and viscous extensibilities (Tanimoto *et al.*, 2000). The freeze–thawing disruption will be increased if the elastic and viscous extensibilities decrease (Satiat-Jeunemaitre *et al.*, 1992; Routier-Kierzkowska *et al.*, 2012). To quantify the extent of the damage to cell structure, we defined the ‘freeze-disrupt coefficient’ (FDC), which was calculated by SA_t_÷SA_c_ (see details in the materials and methods). The larger the FDC is, the more serious disruption occurs in root sections. Therefore, a larger FDC can indicate a greater decrease in the mechanical properties of the cell, indirectly (Tanimoto *et al.*, 2000). In this study, we observed that Al treatment decreased the mechanical properties of root cells. The epidermis and outer cortex cells in Al-treated root sections were damaged and shrunk markedly after a freeze–thawing experiment (Fig. 1E), and the FDC increased significantly (Fig. 1C). Observations by TEM further revealed that the PM turned black, and the CW–PM interface became strongly convoluted after a 6-h exposure to Al (Fig. 2). These results suggested that Al caused a decrease in cells extensibility and increased cell rigidity, resulting in Al-induced inhibition of root elongation (Fig. 1A) and root widening (Fig. 1B). Through studying the effect of Al on viscosity and elasticity of root cell wall by a creep-extension experiment, Ma *et al.* (2004) found that both the viscous and elastic extensibility of cell wall and the “break load” of the root apex were decreased by Al, and, moreover, that these effects were more significant in an Al-sensitive cultivar than in an Al-resistant cultivar. The comparison between *OsPIN2* overexpression lines and wild type showed that the disruption of cells (Fig. 1E), the FDC (Fig. 1C), the root growth inhibition (Fig. 1A and 1B), and the microstructure of PM and CW (Fig. 2) changed less in OXs than in WT under Al stress. These results indicated that overexpressing *OsPIN2* could alleviate Al-induced cell rigidity in the rice root apex, thus relieving the Al-induced inhibition of root growth (Tabuchi and Matsumoto, 2001; Ma *et al.*, 2004).

Mechanical properties belong to the main properties of the cells, and are mainly dependent on basic components, such as the CW, PM, and cytoskeleton (Geitmann and Ortega, 2009). Previous studies indicated that ROS and peroxidase affected the mechanical properties of the cell. An appropriate amount of ROS formation, especially the formation of hydroxyl radicals (·OH) is essential for cell wall loosening (Liszkay *et al.*, 2004). However, a high level of ROS can cause cell wall stiffening and inhibition of cell expansion by inducing wall lignification (Del Carmen Córdoba-Pedregosa *et al.*, 2003; Voothuluru and Sharp, 2013), and damage the integrity of the PM by causing lipid peroxidation and formation of cracks (Yamamoto *et al.*, 2001). According to the content of MDA and lignin in the root apex, we found that both MDA (Fig. 3B) and lignin (Fig. 5A) in WT were obviously increased by Al but not in OX1 and OX2. Furthermore, a stronger Evans blue staining (Fig. 3D) and PM blackening (Fig. 2) was observed in WT than in OX1 or OX2. These results indicated that Al caused more serious oxidative stress in the root apex of WT than that of OXs. The further observations of ROS also verified the above view that less Al-targeted ROS formation occurred in root apexes of OXs than in WT (Fig. 4). Similarly to the results of Sivaguru *et al.* (2013), the Al-induced increase of ROS, including O_2_^–^ and H_2_O_2_ mainly appeared in epidermis and outer cortex (Fig. 4B and 4E). These regions were also easily damaged during the freeze–thawing experiments (Fig. 1E), suggesting that Al-induced increase in ROS may be a significant cause of Al-induced cell rigidity (Yamamoto *et al.*, 2001, 2002; Jones *et al.*, 2006). On the other hand, attenuating Al-targeted oxidative cellular damage may be an important mechanism by which lines overexpressing *OsPIN2* alleviate Al-induced cell rigidity (Krishnamurthy and Rathinasabapathi, 2013*a*).

Antioxidative enzymes such as superoxide dismutase (SOD), ascorbate peroxidase (APX), and catalase (CAT) are served as major ROS-scavenging mechanisms under Al toxicity (Sharma and Dubey, 2007; Matsumoto and Motoda, 2013). According to our further study, we found that all the activities of SOD, CAT, and APX were increased by Al. However, the activities of both SOD and CAT increased more remarkably in OXs than in WT (Fig. S1), which might lead to higher levels of O_2_^–^, H_2_O_2_, and lignification in the root apex of WT than that of OXs.

In the absence of Al, a higher concentration of free IAA was observed in the root apexes of OXs than that of WT (Fig. 7A), suggesting that overexpressing *OsPIN2* regulated IAA transportation in the root apex (Chen *et al.*, 2012) and elevated IAA content in roots. After a short-time exposure to Al, a noticeable difference of increasing IAA concentration was found between WT and OXs (Fig. 7A), indicating that Al inhibited auxin transport, particularly the basipetal auxin transport in root apex, and disturbed the auxin signal transduction (Kollmeier *et al.*, 2000; Sun *et al.*, 2010). However, overexpressing *OsPIN2* might weaken these inhibitory effects of Al. Iglesias *et al.* (2010) found that an *Arabidopsis* auxin signalling mutant, *tir1 afb2* (a mutant for auxin receptor TIR1/AFB proteins) displayed higher activities of antioxidant enzymes and resulted in higher tolerance to oxidative stress than WT, indicating that auxin signalling might participate in the adaptive response against oxidative stress. In this study, we found that overexpression of *OsPIN2* ameliorated an Al inhibitory effect on basipetal auxin transport, suggesting that the effect of Al on auxin signal transduction was weaker in OXs than that in WT. Thus, a higher tolerance to oxidative stress was observed in OXs than WT. Shen *et al.* (2008) showed that Al could disrupt one kind of cytoskeleton proteins, actin microfilament (F-actin), and resulted in a significant inhibition of transport of PIN2 vesicles and polar auxin transport. These results indicated that the interaction of Al with cytoskeleton protein might be one reason to disturb auxin equilibrium (Horst *et al.*, 1999). The different effects of Al on the concentration of free IAA in the root apices of OXs and WT might be associated with different alterations in the cytoskeleton.

Tabuchi and Matsumoto (2001) reported that Al caused hemicellulosic polysaccharides accumulation and resulted in the thickening of the cell wall. Studies in *Arabidopsis* further showed that the lower xyloglucan content in the cell walls resulted in a lower proportion of root cell wall-bounded Al, resulting in less Al-mediated wall tightening (Zhu *et al.*, 2012). Using two rice cultivars differing in Al tolerance, Yang *et al*. (2008, 2013) found that Al treatment resulted in a higher increase in cell wall polysaccharides and pectin methylesterase (PME) activity in an Al-sensitive cultivar than that of an Al-resistant cultivar. In accordance with the results reported by Yang *et al.* (2008), we found that Al treatment resulted in an increased HC1 content in both WT and OXs, and this was more prominent in WT (Fig. 5C). Combining with the Al-targeted oxidative response and the change of CW and PM microstructure, we infer that Al causes the accumulation of ROS, lignin, and HC1, resulting in PM blackening and CW sharpening, and, subsequently, making the cell rigid.

A further examination of Al allocation supported the above hypothesis that a lower proportion of cell wall-bound Al over root sap Al occurred in OXs (Fig. 6C and 6D). These results suggest that overexpressing *OsPIN2* decreases the binding of Al to the cell wall, and such an effect of *OsPIN2* overexpression may be another reason for alleviating Al-induced cell rigidity (Ma *et al.*, 2004; Xia *et al.*, 2010; Zhu *et al.*, 2012). However, it is unknown how OsPIN2 is involved in regulating Al distribution in root cells. Previous studies indicated that operating in concert with OsALS1 (a vacuolar half type ABC transporter), Nrat1 (Nramp aluminium transporter 1) can remove Al^3+^ from the cell wall and sequester it in the vacuole (Xia *et al.*, 2010; Ryan *et al.*, 2011; Huang *et al.*, 2012; Li *et al.*, 2014). Moreover, auxin may regulate Al distribution in cells by altering *ALS1* expression (Zhu *et al.*, 2013). However, no significant differences in the expression of *Nrat1* and *OsALS1* were observed between WT and OX1 (Fig. S2). It seems that *Nrat1* and *OsALS1* make less contribution to the difference in Al content of cell wall and cell sap between WT and OX lines. Other probable mechanisms need to be investigated in the future.

A critical component of the growth-promoting effect by auxin is the acidification of the cell wall via activating PM H^+^-ATPase (Hager, 2003; Grebe, 2005). Studies have shown that auxin and its transporter PIN2 play key roles in root H^+^ secretion and rhizosphere acidification by activating PM H^+^-ATPase (Shen *et al.*, 2006; Xu *et al*., 2012, 2013). In this study, a more significant Al-induced rhizosphere acidification was observed in OXs than in WT (Fig. 7D), suggested that overexpressing *OsPIN2* might increase Al-induced H^+^ secretion. Correlatively, we also found that the abundance of PM H^+^-ATPase genes *OsA1* and *OsA8* in OXs were increased by Al, and only *OsA8* in WT was up-regulated (Fig. 7C), indicating that OXs might have a higher PM H^+^-ATPase activity than WT, and thus have a higher proton-secretion capacity under Al stress (Shen *et al.*, 2005). Combined with the results that IAA is increased by overexpressing *OsPIN2* and Al treatment, we surmised that the increased level and the equilibrium of IAA might cause the higher level of *OSA* expression, and contribute to higher Al tolerance in OXs.

Using the Salkowski colorimetric technique (Glickmann and Dessaux, 1995), we observed an increase of IAA exudation occurring in Al-treated roots, and it was higher in transgenic plants than in wild-type plants (Fig. 7B), which associated with the observation of rhizosphere acidification. Hence, we inferred that this Al-enhanced IAA secretion might contribute to Al-induced rhizosphere acidification, and, further, that this acidification also helped to alleviate Al-induced cell rigidity (Hager, 2003; Grebe, 2005). Future studies are needed to answer the question whether Al-induced IAA exudation is related to vesicular secretion of auxin (Baluška *et al.*, 2008), owing to a growing body of evidence showing that cell-to-cell auxin transport is driven by vesicle recycling (Geldner *et al.*, 2001; Mancuso *et al.*, 2007).

In conclusion, our results indicate that overexpressing *OsPIN2* can alleviate Al-induced cell rigidity in rice root apex owing to less Al-targeted oxidative cellular damage and less Al binding to the cell walls, but more IAA and proton efflux to acidify the cell walls.

## Supplementary data

Supplementary data are available at *JXB* online.

Fig. S1. Effect of Al on the activities of superoxide dismutase (SOD), catalase (CAT) and ascorbate peroxidase (APX).

Fig. S2. Effect of Al on the relative expression of *Nrat1* and *OsALS1*.

Table S1. Primers used in this study.

Supplementary Data

## Abbreviations:

Al: aluminium
IAA: indole-3-acetic acid
PM: plasma membrane
CW: cell wall
ROS: reactive oxygen species
FDC: freeze-disrupt coefficient
