# Supplementary material for: Alleviation of aluminium-induced cell rigidity by overexpression of OsPIN2 in rice roots
Source: J Exp Bot. 2014 Jul 22;65(18):5305–15. doi: 10.1093/jxb/eru292 (PMC4157713; doi:10.1093/jxb/eru292)
Supplement: Supplementary Data [file supp_65_18_5305__index.html]

Alleviation of aluminium-induced cell rigidity by overexpression of OsPIN2 in rice roots — Alleviation of aluminium-induced cell rigidity by overexpression of OsPIN2 in rice roots — Supplementary Data 

# Alleviation of aluminium-induced cell rigidity by overexpression of *OsPIN2* in rice roots

## Supplementary Data

Data files

**Files in this Data Supplement:**

- Supplementary Data - Supplementary Data
