# Supplementary material for: Alleviation of aluminium-induced cell rigidity by overexpression of OsPIN2 in rice roots
Source: J Exp Bot. 2014 Jul 22;65(18):5305–15. doi: 10.1093/jxb/eru292 (PMC4157713; doi:10.1093/jxb/eru292)
Supplement: Supplementary Data [file supp_eru292_jexbot122952_file001.pdf]

Supporting Information

Extended materials and methods

Microscopy observation

3-d-old seedlings of WT, OX1 and OX2 were exposed to a 0.5 mmol L^{-1} $CaCl_2$ solution (pH4.5) containing 0 or 50 μmol L^{-1} $AlCl_3$ for 6 h.

Root tip staining: Root tips (0-10 mm) were excised and used for the histochemical study. The loss of integrity of the plasma membrane was studied by the method of Yamamoto *et al*. (2001) using Evans blue. Root tips were respectively stained in nitroblue tetrazolium (NBT) (Dunand *et al*., 2007) and diamino benzidine (DAB) (Thordal Christensen *et al*., 1997) to analyse the location of superoxide anion (O_2^-) and the hydrogen peroxide (H_2O_2). Al accumulation in root surface was monitored using Eriochrome Cyanine R (Ma *et al*., 2004). All the samples were observed under light microscope (BX43).

O_2^- **located in root tip section:** Roots were washed three times with 0.5 mmol L^{-1} $CaCl_2$ solution, and stained for 10 min in a 0.5% (w/v) NBT solution. After staining, root tips (0-5 mm) were excised, washed with deionized water and embedded in 5% agar, then were transversely sectioned at 3 mm from apexes with vibratome. 50 μm sections were collected and observed under light microscope.

H_2O_2 **located in root tip section:** Root tips (0-5 mm) were excised and embedded in 5% agar, then were transversely sectioned at 3 mm from apexes with vibratome. 50 μm sections were collected and stained in a 10 mmol L^{-1} PBS (pH 7.0) solution containing 1 mg mL^{-1} DAB and 0.1% Triton X-100 overnight. After staining, sections were washed with deionized water and observed under light microscope.

Measurement of physical properties

3-d-old seedlings of WT, OX1 and OX2 were exposed to a 0.5 mmol L^{-1} $CaCl_2$ solution (pH4.5) containing 0 or 50 μmol L^{-1} $AlCl_3$ for 6 h. Root segments (0-10 mm, 60 roots for each replicate) were excised, weighed and used for following physical properties measurement. Protein content was measured by coomassie brilliant blue G-250 method (Bradford, 1976). Lipoxygenase (LOX) activity, superoxide dismutase (SOD) activity, catalase (CAT) activity, and ascorbate peroxidase (APX) activity were analysed according to Aravind and Prasad (2003), Giannopolitis and Ries (1977), Aebi (1984),

and Dalton *et al*. (1987), respectively. The assay of malondialdehyde (MDA) content and lignin content were referred to Heath and Packer (1968) and Fukuda and Komamine (1982), respectively. Cell wall materials were extracted, and uronic acid content in each cell wall fraction was measured according to the method of Yang *et al*. (2008). Amount of H_2O_2 in the root segment samples was quantified as described by Chen and Kao (1999). The rate of O_2^- production was determined according to Elstner and Heupel (1976). The sample preparation and quantification of free IAA were carried out as described by Lu *et al*. (2009). Al content in root apices, root apical cell walls and cell sap was examined according to Huang *et al*. (2012).

Gene expression

3-d-old seedlings of WT, OX1 and OX2 were exposed to a 0.5 mmol L^{-1} $CaCl_2$ solution (pH4.5) containing 0 or 50 μmol L^{-1} $AlCl_3$ for 6 h. Root apices (0-10 mm, 30 root each) were collected for RNA extraction using E.Z.N.A.® Plant RNA Kit (Omega, USA.). The first-strand cDNA was synthesized with PrimeScript® RT Master Mix (TaKaRa, Japan). Semi-quantitative RT-PCR was performed to analysis *OsA* family gene expression using the gene-specific primers (Table S1) according to Chang *et al*. (2009), and *Histone H3* (Huang *et al*., 2012) was used as loading control. Real-time RT-PCR was performed to analysis *Nrat1* and *OsALS1* expression using the gene-specific primers (Table S1) according to Huang *et al*. (2012), and *Histone H3* was used as internal reference gene.

References

Aebi HE. 1984. Catalase in vitro. *Methods in Enzymology* **105**, 121–126.

Aravind P, Prasad MNV. 2003. Zinc alleviates cadmium-induced oxidative stress in *Ceratophyllum demersum* L.: a free floating freshwater macrophyte. *Plant Physiology and Biochemistry* **41**, 391–397.

Bradford MM. 1976. A rapid and sensitive method for the quantitation of microgram quantities of protein utilizing the principle of protein-dye binding. *Analytical Biochemistry* **72**, 248–254.

Chen L, Kao C. 1999. Effect of excess copper on rice leaves: evidence for involvement of lipid peroxidation. *Botanical Bulletin of Academia Sinica* **40**, 283–287.

Dalton DA, **Hanus FJ**, **Russell SA**, **Evans HJ.** 1987. Purification, properties, and distribution of ascorbate peroxidase in legume root nodules. *Plant Physiology* **83**, 789–794.

Elstner EF, Heupel A. 1976. Inhibition of nitrite formation from hydroxylammoniumchloride: a simple assay for superoxide dismutase. *Analytical Biochemistry* **70**, 616–620.

Fukuda H, Komamine A. 1982. Lignin synthesis and its related enzymes as markers of tracheary-element differentiation in single cells isolated from the mesophyll of *Zinnia elegans*. *Planta* **155**, 423–430.

Giannopolitis CN, Ries SK. 1977. Superoxide dismutases: I. Occurrence in higher plants. *Plant Physiology* **59**, 309–314.

Heath RL, Packer L. 1968. Photoperoxidation in isolated chloroplasts: I. Kinetics and stoichiometry of fatty acid peroxidation. *Archives of Biochemistry and Biophysics* **125**, 189–198.

Lu YL, Xu YC, Shen QR, Dong CX. 2009. Effects of different nitrogen forms on the growth and cytokinin content in xylem sap of tomato (*Lycopersicon esculentum* Mill.) seedlings. *Plant and Soil* **315**, 67–77.

Supporting figures

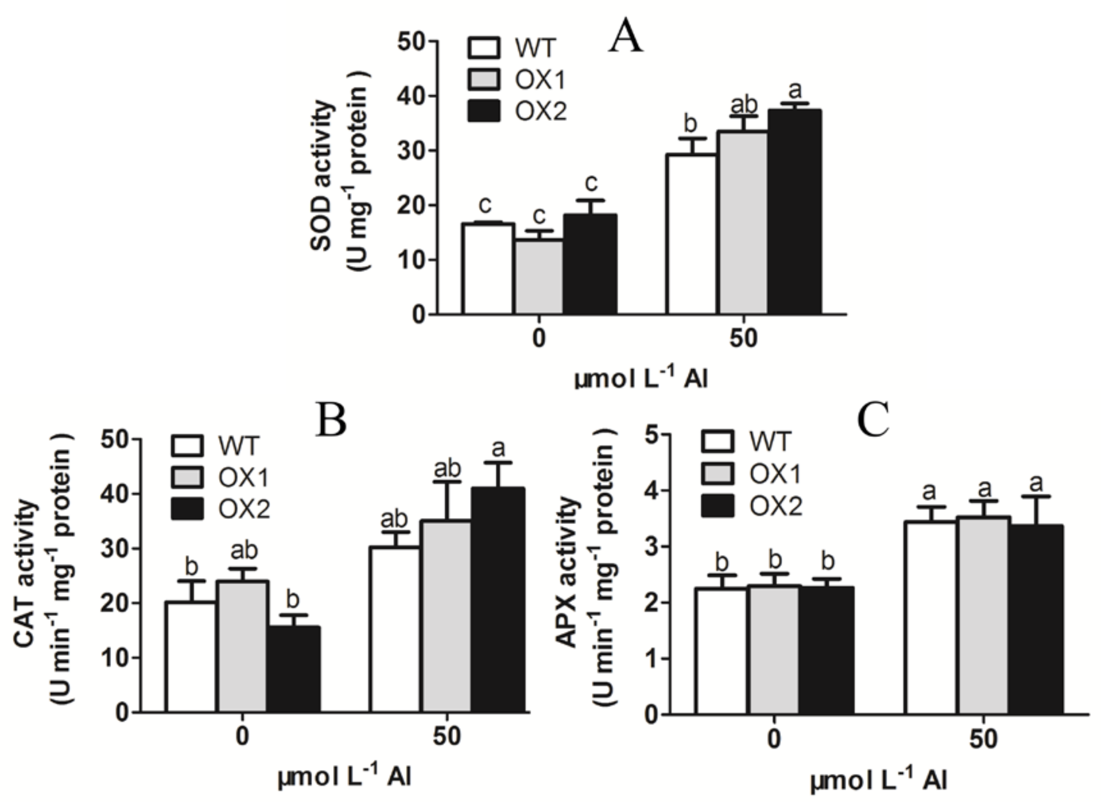

Fig. S1. Effect of Al on the activities of superoxide dismutase (SOD, A), catalase (CAT, B) and ascorbate peroxidase (APX, C). Values are mean ± SE (n = 3). Different letters above the column indicated significant differences (P<0.05 by Tukey test).

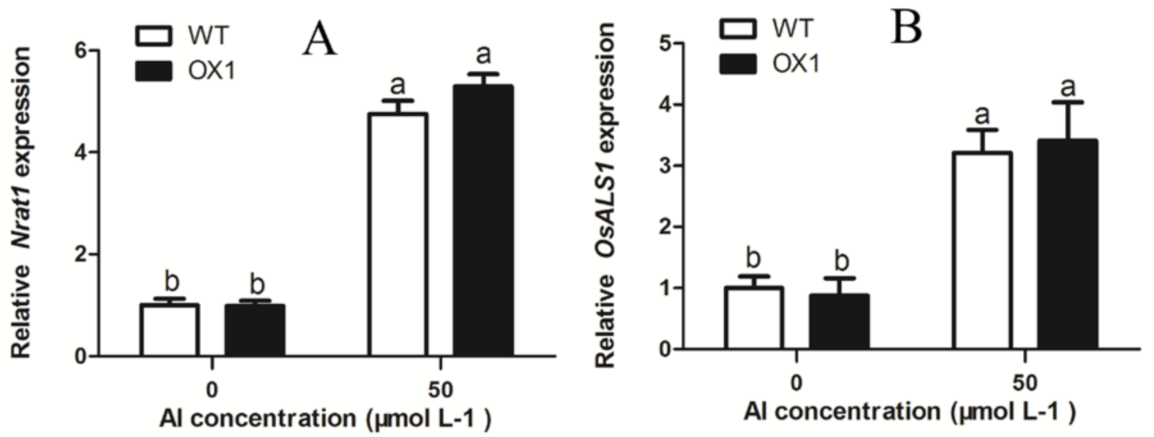

Fig. S2. Effect of Al on the relative expreession of *Nrat1* (A) and *OsALS1* (B). Values are mean ± SE (n = 3). Different letters above the column indicated significant differences (P<0.05 by Tukey test).

Table S1 Primers used in this study

Gene	Gene ID	Primers
OsA1	Os03g0689300	F: 5-TGGCTGGCATGGATGTTCTT-3
		R: 5-TTCCTAGACGACGCCCTGTTT-3
OsA2	Os07g0191200	F: 5-TTGCCATGCCCACTGTTCTT-3
		R: 5-GCGTGCTGTTTCCTTGCCTAT-3
OsA3	Os12g0638700	F: 5-CGGAGATAGAGCGGAGGGT-3
		R: 5-CGACGCCCTGTTTCTTTTCC-3
OsA7	Os04g0656100	F: 5-GCCATGCCTACCGTGCTCTC-3
		R: 5-CCCATTCCAAGCCTCCTACCA-3
OsA8	Os03g0100800	F: 5-TCAGTTGGCTATTGGTAAGG-3
		R: 5-ATGGTGCTCACTTGAAGGT-3
Nrat1	Os02g0131800	F: 5-GAGGCCGTCTGCAGGAGAGG-3
		R: 5-GGAAGTATCTGCAAGCAGCTCTGATGC-3
OsALS1	Os03g0755100	F: 5-GGTCGTCAGTCTCTGCCTTGTC-3
		R: 5-CCTCCCCATCATTTTCATTTGT-3
Histone H3	Os06g0802700	F: 5-AGTTTGGTCGCTCTCGATTTCG-3
		R: 5-TCAACAAGTTGACCACGTCAC-3
